# Changes in Clinical Training for Nursing Students during the COVID-19 Pandemic: A Scoping Review Protocol

**DOI:** 10.3390/nursrep12010021

**Published:** 2022-03-14

**Authors:** Catarina Lobão, Adriana Coelho, Rui Gonçalves, Vitor Parola, Hugo Neves, Joana Pereira Sousa

**Affiliations:** 1 Health Sciences Research Unit: Nursing (UICISA: E), Nursing School of Coimbra (ESEnfC), 3000-232 Coimbra, Portugal; adriananevescoelho@esenfc.pt (A.C.); rgoncalves@esenfc.pt (R.G.); vitorparola@esenfc.pt (V.P.); hugoneves@esenfc.pt (H.N.); 2 Portugal Centre for Evidence-Based Practice: A Joanna Briggs Institute Centre of Excellence, 3000-232 Coimbra, Portugal; joana.sousa@ipleiria.pt; 3 Center for Innovative Care and Health Technology—ciTechCare, School of Health Sciences-Polytechnic of Leiria, 2411-091 Leiria, Portugal

**Keywords:** changes, clinical training, COVID-19, nursing students, review

## Abstract

**Background**: The COVID-19 pandemic has had consequences for social, economic, cultural and educational life, affecting nursing training and practice. To date, no previous scoping reviews addressing this objective have been found. This study aims to map the literature related to changes in clinical training for nursing students during the COVID-19 pandemic. **Methods**: A scoping review will be carried out according to the Joanna Briggs Institute’s latest guidance regarding methodology. A set of relevant electronic databases and grey literature will be searched using terms such as clinical practice, nursing students, COVID-19. **Results**: This scoping review will consider any type of quantitative, qualitative, and mixed-methods study and systematic review designs for inclusion, focusing on changes in clinical training for nursing students during the COVID-19 pandemic. **Conclusion**: Pedagogical criteria had to be changed due to the COVID-19 pandemic, especially face-to-face clinical training for nursing students. Identifying the changes in clinical training for nursing students during the COVID-19 pandemic will help educators to understand the potential impact of this specific context and trace possible gaps. This protocol is registered at Open Science Framework.

## 1. Introduction

The emergence of the SARS-CoV-2 coronavirus and its rapid spread worldwide prompted the World Health Organization to declare a pandemic state on 11 March 2020. Changes were required in world dynamics and society in general to combat the spread of the new coronavirus [1].

There was a need to reinvent strategies and readapt the teaching, learning, and assessment processes in nursing education [2,3], through digital training programs or tools such as simulation and telehealth [4,5].

The nursing discipline focuses on human responses to health-disease phenomena and life processes, with face-to-face nursing care being essential [6]. Thus, the training of health professionals to take care of people requires developing skills resulting from the action and articulation of various actors, encouraging debate, exchanging experiences, interaction, reflection, and critical thinking [6].

The impact of the new coronavirus has created unusual learning methods for nursing students. The clinical placement can be experienced as a challenging part of training, even discounting from the pandemic situation. Students already struggle to be part of a care team, where professional self is not yet defined, leading to feelings of insecurity about their competence [7].

The pandemic has raised several challenges in teaching nursing students, namely in the clinical context. Uncertainty about the reception of students in healthcare teams or even the interruption of clinical training enhanced the need for a solution to promote clinical training by means of a simulation interface [7,8].

Additionally, students could not develop their practical activities in the clinical context at pre-licensing and advanced practice levels. This phenomenon required ingenious solutions to promote students’ training, allowing them to complete their training programs at the usual schedule [8].

Training nursing students in a pandemic context is an urgent need. However, many clinical settings have interrupted or postponed the nursing students’ clinical training due to lockdown policies, scarcity of material (specifically, individual protection equipment (IPE)), workload-related burnout, and the obligation to reduce the movement of people in clinical practice care settings [4,9]. Nevertheless, final year undergraduates have contributed to the fight against the pandemic in many contexts. This reality allowed for continuing the learning processes in clinical education by integrating the health teams created to respond to the pandemic [4,9]. However, while some students participating in this catastrophic scenario saw this as an extremely attractive challenge, which allowed knowledge consolidation in a historical era, the challenge has been seen as demanding and painful by others [9].

Despite recognizing the challenges that the pandemic has created, in clinical internships, nursing students revealed understanding and acceptance of the needed change. On the other hand, students mentioned that it was difficult to find an inbound clinical setting [7,8], which influenced their ability to adapt to this new reality, personally and academically. The need for an adjustment is reflected in students’ achievements and expectations [3], based on their wellbeing [10,11,12], stress levels [13] and perception of their quality of life [14].

In all graduation levels, students will play a crucial role in future pandemics. When students are not adequately prepared in the art of care, simulation training improves anxiety and stress levels, especially in the simulation on managing critical patients and ventilatory support [15].

This scoping review is guided by the Joanna Briggs Institute’s (J.B.I.) methodology to conduct scoping reviews, and aims to map the changes in clinical training for nursing students during the COVID-19 pandemic. An initial search of MEDLINE (PubMed), the J.B.I. Evidence Synthesis, the Cochrane Database of Systematic Reviews, PROSPERO, and Open Science Framework (O.S.F.) revealed that currently, there are no scoping reviews or systematic reviews (published or in progress) about this subject [16,17,18].

The main goal of this scoping review is to map the changes in clinical training for nursing students during the COVID-19 pandemic. It can significantly contribute to understanding this phenomenon to aid nursing educators in developing programs and proposals to target clinical teaching, learning, and assessment strategies for nursing students in similar contexts. This map will identify relevant topics to assist in advancing evidence-based nursing education, develop knowledge, and identify potential gaps.

This scoping review seeks to answer the following questions:

- What are the changes in clinical training for nursing students during the COVID-19 pandemic? (e.g., contamination risk; IPE);
- What is the context of clinical practice training for nursing students where the changes are described? (e.g., clinical training services);
- What are the educational implications of nursing students’ learning processes reported? (e.g., postponement, withdrawal, interruption).

## 2. Materials and Methods

The protocol for this scoping review will be guided following the J.B.I.’s latest guidance regarding methodology [16,17,18]. The final review will be reported following the Preferred Reporting Items for Systematic Reviews and Meta-Analyses extension for Scoping Reviews (PRISMA-ScR) guidelines [19]. This review protocol was registered in the Open Science Framework (https://osf.io/mduve/ (accessed on 21 April 2021)).

### 2.1. Inclusion Criteria

Based on the J.B.I. recommendations regarding the mnemonic “P.C.C.” for scoping reviews, the inclusion criteria will include: participants—this review will consider studies that include undergraduate nursing students; concept—this review will consider studies exploring nursing students’ clinical training changes during the COVID-19 pandemic; context—this review will consider studies, independently of the country of the study, conducted in any clinical practice setting; and types of sources—this scoping review will consider any quantitative, qualitative, mixed methods study designs, editor letters and guidelines for inclusion. Additionally, all types of systematic reviews will be considered for inclusion in the proposed scoping review.

### 2.2. Search Strategy

The search strategy will locate both published and unpublished primary studies and reviews.

Two reviewers developed the search strategy, which was peer-reviewed by the expert third reviewer considering the Peer Review of Electronic Search Strategies (PRESS) checklist [20]. The J.B.I.’s recommended three-step search strategy will be applied [16,18]. A limited preliminary search was undertaken on MEDLINE (via PubMed) and CINAHL Complete (EBSCOhost) to find articles on the topic. Thus, the text words in the titles and abstracts of pertinent articles and the index terms used to describe the articles were used to create a full search strategy for MEDLINE (via PubMed), as seen in Table 1. The search was conducted on 17 January 2022. The search strategy will be adapted to the specificities of each information source. Lastly, the reference lists of the articles included in the review will be screened for supplementary papers.

Study languages will be restricted to those mastered by the authors—English, Spanish and Portuguese—in order to ensure a good-quality selection procedure and data extraction. Document studies in other languages, excluded based on language, will be stated for transparency in the scoping review report.

The databases to be searched will include MEDLINE (via PubMed), CINAHL complete (EBSCOhost), Cochrane Central Register of Controlled Trials, Cochrane Database of Systematic Reviews, LILACS, Scopus, and scientific libraries, such as SciELO. The search for unpublished studies will include DART-Europe; OpenGrey or other grey literature (e.g., Editor letters or guidelines).

### 2.3. Study Selection

All of the records identified during the database search will be retrieved and stored in the Mendeley^®^ V1.19.4 (Mendeley Ltd., Elsevier, Amsterdam, The Netherlands), and duplicates removed. Two reviewers will independently screen the titles and abstracts. A pilot test will be undertaken to verify whether inclusion criteria are met. Potentially eligible studies will be assessed according to whether the full text is available, whether they meet the inclusion criteria, whether the abstract is unclear, and whether the study’s relevance is uncertain, while their citation details will be imported into the J.B.I. System for the Unified Management, Assessment and Review of Information (JBI SUMARI; J.B.I., Adelaide, Australia) [21]. Secondly, the full text of selected citations will be assessed in detail, against the inclusion criteria, by the two independent reviewers. Full-text studies will be excluded if they do not meet the inclusion criteria. In addition, the reasons for exclusion will be provided in an appendix in the final report of the scoping review. Finally, the references of all the included studies in the review will be hand-searched. Disagreements between the two reviewers will be resolved through discussion or with a third reviewer at each stage of the selection process. In the case of an inaccessible full article, the author will be contacted.

The search results will be detailed in the final scoping review and presented in a Preferred Reporting Items for Systematic Reviews and Meta-analyses for Scoping Reviews (PRISMA-ScR) flow diagram [19].

### 2.4. Data Extraction

Extracted data from included articles will be charted according to the J.B.I.-proposed template by the two independent reviewers [16,18] and aligned with the goals and research questions. A draft extraction tool is presented in Table 2. The draft data extraction tool could be revised as necessary during data extraction from each included paper. Levac, Colquhoun and O’Brien [22] suggested that to ensure consistency of data extraction, a priori pilot charting of the first five to ten studies should be made by two reviewers, independent of each other. The decision of a third reviewer will solve any disagreements in data extraction.

Study authors will be contacted for further data information in the case of missing data. Because review studies will be included, reviewers will choose to report the preliminary study in the case of data duplication.

### 2.5. Data Analysis and Presentation

The data collected will be shown in tabular form (Table 3), depending on which is more appropriate to this review’s objective. A descriptive summary will be provided regarding the charted result aligned with this scoping review’s purpose [16,18] and a qualitative coding might emerge from the data analysis.

## 3. Discussion

This scoping review will only consider English, Portuguese, and Spanish studies, which can be registered as a potential study limitation. To overcome this limitation, abstracts of articles published in other languages, which could also be important to include in this review, will be translated through Google Translator and Linguee to prevent restricting ourselves to programs specific to certain cultures.

## 4. Conclusions

We believe that the academic community has reflected on the changes driven by the COVID-19 pandemic. Thus, this scoping review will allow pedagogical structures to embrace the strategies arising from these findings to establish programs that support clinical training for undergraduate nursing students. This scope will also identify possible gaps in future research work.

## Figures and Tables

**Table 1:** Search strategy for MEDLINE (via Pubmed).

Search	Query	Record Retrieved
#1	“nursing students”[Title/Abstract] OR “nursing student”[Title/Abstract] OR “nurse students”[Title/Abstract] OR “nurse student” [Title/Abstract] OR “students, nursing”[MeSH Terms]	34,097
#2	“clinical training”[Title/Abstract] OR “clinical learning”[Title/Abstract] OR “clinical practice”[Title/Abstract] OR “preceptorship”[MeSH Terms] OR “Preceptorship”[Title/Abstract]	228,818
#3	“COVID-19”[MeSH Terms] OR “COVID-19”[Title/Abstract] OR “Sars-CoV-2”[Title/Abstract] OR “Sars-CoV-2”[MeSH Terms]	212,741
#4	((“nursing students”[Title/Abstract] OR “nursing student”[Title/Abstract] OR “nurse students”[Title/Abstract] OR “nurse student”[Title/Abstract] OR “students, nursing”[MeSH Terms]) AND (“clinical training”[Title/Abstract] OR “clinical learning”[Title/Abstract] OR “clinical practice”[Title/Abstract] OR “preceptorship”[MeSH Terms] OR “Preceptorship”[Title/Abstract])) AND (“COVID-19”[MeSH Terms] OR “COVID-19”[Title/Abstract] OR “Sars-CoV-2”[Title/Abstract] OR “Sars-coV-2”[MeSH Terms])	67

**Table 2:** Data extraction tool.

Scoping Review Details	
Scoping review title	Changes in clinical training for nursing students during the COVID-19 pandemic: a scoping review protocol
Review objective(s)	Map the changes in clinical training for nursing students during the COVID-19 pandemic situation.
Review question(s)	What are the changes seen in clinical training for nursing students during the COVID-19 pandemic. What are the nursing students’ perceptions about the changes in clinical training during the COVID-19 pandemic (exploring causal factors); What are the contexts of nursing students’ clinical training where the changes are observed (context of learning/clinical training services); What are the implications of the changes to nursing students’ learning processes (academic and personal; postponement, withdrawal, interruption).
Inclusion/Exclusion Criteria	
Population	This review will consider studies that include undergraduate nursing students.
Context	This review will consider studies conducted in any clinical practice setting.
Concept	This review will consider studies that explore changes and challenges in clinical training for nursing students during the COVID -19 pandemic.
Types of evidence source	This scoping review will consider any quantitative, qualitative, and mixed methods study designs for inclusion. Additionally, systematic reviews will be considered for inclusion in the proposed scoping review.
Evidence Source Details and Characteristics	
Author(s)	
Year of publication	
Origin/country of origin (where the source was published or conducted)	
Aims/purpose	
Population and sample size	
Details/Results Extracted from the Source of Evidence (concerning the concept of the scoping review)	
Changes and challenges in clinical training	
Perception of nursing students	
Context of in clinical training	
Academic implications	
Personal implications	

**Table 3:** Data collection in tabular form.

	Study 1	Study 2	…	…
Changes in clinical training				
Context of clinical training				
Academic implications				
Personal implications				

## Data Availability

Not applicable.
